# The Ancients on Equine Age Marks

**Published:** 1884-01

**Authors:** William H. Clarke


### Art. III.—THE ANCIENTS ON EQUINE AGE MARKS.

BY WILLIAM H. CLARKE.

IN the October, 1882, number of the Journal Dr. Robert Jennings spoke of the ancients being aware of the age-marks in horses' teeth, and mentioned several well-known ancient authors in proof of what he said. Dr. Jennings is right, except that two of the authors he named (Hippocrates and Xenophon) do not sustain him. During the revision of my work on horses' teeth I consulted many ancient authors. Some of them not only understood the value of the marks, but were aware of the fact that the twelve first molars are shed, notwithstanding the assertion of Aristotle to the contrary. With your permission I will give the testimony of a few of the ancients about horses' teeth, giving the authors in their chronological order.

Xenophon (B. C. 444) gives several rules for bridling a horse. The last one is as follows: "And if he does not receive it then, let the lip be pressed to the eye-tooth. There are very few which do not receive it when they suffer this." Again, in giving rules for purchasing a horse, "to avoid being cheated in the bargain," he says: "First, then, let it not escape notice what his age is, for if he has not the foal-teeth he can neither give us pleasure with anticipated exertion, nor can be easily disposed of again."—[*Fieldings' Trans., p.* 719.

Aristotle (B. C. 384) says (*His. Animals, Bohn's Trans. pp.* 170-1 :)

"The horse and the mule attain perfection after casting their teeth, and when they have cast them all it is not easy to know their age. Wherefore, they say that before casting its teeth the horse has its mark, which it has not afterward. After the teeth have been changed, the age is usually ascertained by the canine tooth, for that in riding horses is generally worn down, for the bridle rubs against it. In horses which have not been ridden it is large and not worn. In young horses it is small and sharp. * * * The ass sheds its first teeth at thirty months old. The second six months afterward. The third and fourth in the same way. These fourth teeth are called the marking-teeth."

In the case of Aristotle the inference is that he understood the nature of the marks, but his language is contradictory, for the marks in the second set of teeth are larger than those of the first. Of course the artificial marks of the bit-worn tushes do not count.

Varro (B. C. 116) says: "Should they desire to form herds of horses and mares, such as are seen in the Peloponnese and in the Appulia, they should, first of all, ascertain the age of the individuals, which, it is said, must not be less than three nor more than ten years old. It is by the teeth that they find out the age of a horse, as well as that of all split-footed animals. When two years and a half old the horse begins to lose his four middle teeth (two upper and two lower). On entering his fourth year he loses again (from each jaw) the two next to those he has already shed, and then the two large teeth, called molars, begin to grow. When he reaches his fifth year he loses again two others in the same way. Others grow in their place, which, hollow at first, begin to fill up in the sixth year, so that when he is seven years old the animal has his set complete. From that time on there are no sure indications of age, only when the teeth project out of his mouth, when his eyebrows are white, and when his hollow pits cave in under his eyebrows, they suppose he is sixteen years old.—[*Book II., Cap. VII.**

This is an excellent description of the eruption and shedding process, and the first clear description of the marks I have found. Varro was a practical as well as a learned man. "The origin of a horse," says he, "is a matter of the greatest importance, as there are so many species." Again, speaking of veterinary science. "The multitude of the maladies and the diversity of the symptoms render the science very complicated, and, to prevent mistakes, it is indispensable that prescriptions should be written."

Columella (A. D. 42) says: "A horse may be broken for domestic uses when two years old; but when intended for war he must be over three years, so that he may not be exposed to it before he has accomplished his fourth year. The marks by which they distinguish the age of a horse change with his body. Thus, when he is two years and a half old, the middle teeth—upper as well as lower—fall off. Others grow again in the fourth year, after those that are called canine, or eye-teeth, have fallen off. Afterward the superior molars fall before the sixth year. In the course of the sixth year those that have replaced the first ones fill up, and by the seventh year all are full evenly. Afterward they dig in, and the age can no longer be told with certainty. However, at his

---

* Archidemus, a very ancient Greek, is supposed to be the author of a few fragments of veterinary science that have been preserved in the *Veterinariæ Medicinæ, Libri Duo*, by J. Ruellius, Paris, 1530, a book that was compiled by order of Constantine VII., which also contains fragments from the works of Apsyrtus and other authors, and which is pronounced by A. Rambaud (Paris 1870) to be a "celebrated collection." Apsyrtus (or Absyrtus, as the name is sometimes written) flourished about A. D. 330. He described glanders, fevers, epizootic influenza, dental cysts, etc. He is said to be "the principal veterinary surgeon of whom any remains are still extant."

tenth year his temples begin to cave in, his eyebrows often become white and his teech protrude out of his mouth."—[*Book VI, Cap. XXIX.*]

Columella, so far as I know, was the first to describe the shedding of the molars. In Latin he says: "*Intra sextum deinde annum, molares superiores cadunt.*"* Like Varro and others, he says the teeth "fill up," a mistake that is common to this day. The cavities (marks), unlike the pulp cavities, do not fill up with tooth substance; they are obliterated by wear.

Palladius, who, like Varro and Columella, wrote much on agriculture, understood the marks and the shedding of the grinders too. His date is uncertain, but he probably flourished about A. D. 400. He says:

"Here are the signs by which the age of horses is known: When two years and a half (old) they lose their upper middle teeth; at four years their canines change; before the sixth year their upper molars fall off; in the course of the sixth year those teeth that changed first fill up, and at the seventh year they are all full. Past that age there are no longer any sure signs of their age unless this: that when they are advanced in years their temples begin to cave in, their eyebrows become white and their teeth usually protrude."—[*Book IV, Cap. XIII.*

Vegetius Renatus Publius, (often confounded with Vegetius Renatus Flavius, a military writer,) flourished, probably, about A. D. 400 or 500. As he quotes from the works of Apsyrtus, it is plain he was either contemporary with, or lived after, Apsyrtus' time. It is clear that he understood that the first twelve molars are shed, for he says, "the grinders disappear." His description of the auxiliary signs of age, so far as it goes and so far as I know, has never been surpassed. The language, however, is not elegant. He says:

"The age of beasts of burden can be known from their teeth, and buying we should neither submit to the disadvantage of the ignorant, nor curing the sick must we be ignorant of the age. Because, as with men, so also with horses; one thing is proper when they are high-spirited, another when frigid with age. Moreover, it is manifest that the marks of the body are changed with age; for, with young animals two and a half

---

* The upper molars fall about the sixth year.

years old, the middle upper teeth, which they call milk teeth, fall out; but when they begin to reach the fourth year, these falling off, others take their place, which are called dog teeth. Then within the sixth year the grinders disappear. In the sixth year those that are equal change first. In the seventh year all are equally filled out, and from this time they begin to have the dog teeth.* Age is not hereafter known to a certainty unless by other signs which usage teaches. In the tenth year the temples begin to whiten and the eyebrows sometimes become gray. In the twelfth year a blackness in the middle of the teeth appears. Frequently they scatter wrinkles on the upper lips of domestic animals and those accustomed to the bit, so that from the corner where the wrinkles begin we go even to the end of the lip, because the number of wrinkles shows the number of years. Finally, age is shown by the number of wrinkles, the sadness of the countenance, the dejection of the neck, the stupor of the eyes, the baldness of the eyelids and the lassitude of the whole body."—[*Book IV, Cap. V.*

So it is clear that the discoveries of Ruini and Tenon in the sixteenth century and that of Pessina in the nineteenth, while they may have been entirely honest, were not *first* discoveries. The fame of Aristotle caused his mistake to be so deeply rooted that the comparatively obscure but intelligent subsequent authors could not, or at least did not correct it. Men hear of obscure authors, but they rarely read them.

Pliny (B. C. 23) compiled from many authors, and correctly described the eruption and shedding of the incisors; but, while he repeated Aristotle's mistake about the non-shedding of the molars, he failed to give the latter's description (imperfect, of course) of the marks, and also that of both Varro and Columella. But it is possible that he never saw *all* of Columella's works, as the men were contemporary; but he quotes from him extensively.

Here is a good point from Pliny about the human teeth:

"It is the office of the front teeth to regulate the voice and the speech. By a certain arrangement they receive, as if in concert, the stroke communicated by the tongue, while by their

* By dog teeth he probably means the smaller and sharper teeth, caused by change in shape.

structure in such regular order, and their size, they cut short, modulate, or soften the utterance of the words, when they are lost the articulation becomes altogether confused and indistinct."

Some of the following statements from Pliny are erroneous and some are extraordinary, to say the least (Vols. II. and III.):

"There is no animal that changes the maxillary teeth which stand beyond the canine teeth. While in all other animals the teeth grow of a tawny color with old age, with the horse, and him only, they become whiter the older he grows. If a horse is gelded before it changes its teeth, it never sheds them. A female ass, which has not conceived before shedding what are called the milk teeth, is considered to be barren. It is said that Cæsar, the Dictator, had a horse which would allow no one to mount but himself, and that his forefeet were like those of a man. Indeed it is thus represented in the statue before the Temple of Venus Genetrix. Some persons are born with a continuous bone in the mouth in place of teeth. This was the case of the upper jaw of a son of Prusias, the King of Bithynia. The teeth are the only parts of the body that resist the action of fire. Still though they are able to resist the action of flame, they become corroded by a morbid state of the saliva. The canine teeth, when lost by an accident, are never known to come again. Mucianus has stated that he himself saw one Zocles, a native of Samothrace, who had a new set of teeth when he was past his one hundred and fourth year. The number of teeth allotted to all men, with the exception of the nation of the Turduli, is thirty-two. In the human teeth thera is a certain venom; for if they are placed uncovered before g mirror they will tarnish its brightness, and they will kill youn pigeons while yet unfledged. In addition to these facts, in man males have more teeth than females, which is the case also n sheep, goats and swine."

